# Dendritic beading during early brain development impairs signal transmission and synaptic plasticity

**DOI:** 10.1186/s40478-025-02123-8

**Published:** 2025-10-06

**Authors:** Pratyush Suryavanshi, Satya Murthy Tadinada, Samuel Baule, Naisha Jhaveri-Cruz, Ted Abel, Joseph Glykys

**Affiliations:** 1 Department of Pediatrics, Carver College of Medicine, University of Iowa, Iowa City, IA 52242 USA; 2 Iowa Neuroscience Institute, University of Iowa, Iowa City, IA 52242 USA; 3 Department of Neuroscience and Pharmacology, University of Iowa, Iowa City, IA 52242 USA; 4 Department of Biomedical Engineering, University of Iowa, Iowa City, IA 52242 USA; 5 Department of Neurology, Carver College of Medicine, University of Iowa, Iowa City, IA 52242 USA

**Keywords:** Dendritic blebbing, Neonatal, Status epilepticus, Cognitive impairment, Early-life seizures, Brain development

## Abstract

Excessive glutamate receptor activation during brain pathologies causes varicose dendritic swelling, also known as “dendritic beading”, yet its impact on developing brain circuits is poorly understood. Using field electrophysiology and two-photon imaging in awake, behaving mice and acute brain slices (P11–19), we found that severe and recurrent seizure-like activity (induced by NMDA and 4-aminopyridine) resulted in widespread, long-lasting dendritic beading and spine loss in cortical and hippocampal neurons, with localization patterns distinct from those described in adults. Beads showed persistently high calcium levels and stopped the spread of dendritic calcium signals. Dendritic beads suppressed hippocampal evoked field potentials, followed by only partial recovery, and reduced hippocampal long-term potentiation. Clinically used hyperosmotic treatments (mannitol or hypertonic saline) reduced seizure-induced beading and restored dendritic signal propagation. These findings suggest that seizure-induced dendritic beading disrupts circuit function and synaptic plasticity and may contribute to cognitive deficits after early-life seizures.

**Supplementary Information:**

The online version contains supplementary material available at 10.1186/s40478-025-02123-8.

## Introduction

Hyperexcitability during early brain development is often associated with cognitive deficits later in life [79]. Cognitive impairment and intellectual disability (ID) are common in newborns who suffer severe and intractable seizures [56, 78, 80]. Similarly, other excitotoxic insults, such as hemorrhagic strokes or hypoxic-ischemic insults during early development, result in significant cognitive impairments in humans and animal models [40, 47, 85]. These cognitive dysfunctions are often attributed to brain lesions and cell death [41, 48]. However, clinical and experimental evidence also hint at cognitive defects without apparent lesions or substantial neuronal loss following early-life excitotoxicity [4, 58]. There is a critical need to understand the impact of “non-lethal” forms of injury that induce long-term alterations in morphology, synaptic transmission, and network processing.

One relatively unexplained “non-lethal” consequence of several brain insults is the varicose swelling (beading) of neuronal dendrites, often accompanied by the loss of dendritic spines [22, 72]. Dendritic beading occurs during various excitotoxic brain insults, including severe seizures, spreading depolarizations, and hypoxic-ischemic injury, among others [17, 67, 87], primarily due to the excessive stimulation of NMDA and other glutamate receptors [28, 30, 46]. Experimental evidence from adult mice suggests that dendritic beading is often a harbinger of neuronal injury, with beading appearing first in distal dendrites and then progressively in proximal segments, suggesting a continuous injury [29, 73]. Furthermore, observations from human tissue and animal models of focal intractable epilepsy suggest that dendritic beading is also associated with a marked loss of spines [12, 72]. Hence, dendritic beading and the resultant spine loss could be catastrophic for normal circuit function and plasticity mechanisms, especially during early brain development, which may contribute to cognitive deficits. However, the precise changes in dendritic morphology induced by early developmental excitotoxicity and its effects on neural circuit function remain relatively unexplored.

In this study, we examined the morphological and functional effects of dendritic beading resulting from excitotoxicity during early brain development. Using multiphoton microscopy in transgenic mice with sparse neuronal expression of YFP and GCaMP6s, we imaged the effect of excitotoxic injury on dendritic morphology and stimulus-evoked Ca^2+^ transients at high spatiotemporal resolution. We also evaluated the impact of common clinical treatments for brain edema on beading-induced disruption of dendritic function. Next, we assessed the impact of early developmental excitotoxicity and the resulting dendritic beading on hippocampal synaptic plasticity, a physiological correlate of learning and memory. Our findings shed light on how non-lethal dendritic beading results in altered long-term potentiation, which may underlie cognitive deficits associated with disorders of neuronal hyperexcitability. Our results also highlight the importance of mitigating non-lethal dendritic injury as a potential avenue for improving dendritic signal transmission and hippocampal plasticity following early-life seizures and other excitotoxic insults.

## Methods

### Animals and study design

All experiments were conducted on male and female mouse pups using a protocol approved by The University of Iowa's Institutional Animal Care and Use Committee. All mice were housed in a temperature and humidity-controlled vivarium with free access to food and water, maintained on a 12-h light/dark cycle. We used transgenic mice that sparsely express a genetically encoded stable yellow fluorophore in neurons (Thy1-YFP-H, Jax #003782) or the Ca^2+^ indicator GCaMP6s (Thy1-GCaMP6s/GP4.3, Jax #024275). We also used C57BL/6J mice (Jax #000664). Acute brain slices were prepared using hemizygous and homozygous postnatal days 11–19 (P11–19) pups (49 pups used for the study). Experiments on acute brain slices were performed using male and female mouse pups (P11–13, n = 33). For hippocampal LTP experiments, we used acute brain slices prepared from slightly older male and female mice (P15–19, n = 10). We conducted awake, in vivo imaging experiments on P14–17 pups (n = 6). Table 1 in the additional file has the description of the mouse ages for each experiment. The sample size was determined based on previous reports and power analysis. Although both sexes were used for experiments, there was no observable difference in excitotoxic dendritic dysfunction between male and female mice.

### Preparation of acute brain slices

Pups were anesthetized using isoflurane inhalation and decapitated as described previously [19]. Briefly, each brain was removed and placed in ice-cold artificial cerebrospinal fluid (aCSF) containing (in mM) NaCl (116), KCl (3.3), CaCl_2_ (1.3), NaH_2_PO_4_ (1.25), NaHCO_3_ (25), and D-glucose (10) and mannitol (20) along with a high MgCl_2_ concentration (2) and kynurenic acid (2) to block glutamatergic receptors (osmolarity: 300 mOsm). The aCSF was saturated with carbogen (95% O_2_ and 5% CO_2_) to maintain a pH of 7.3–7.4. Thick coronal sections (450 µm) containing the sensory neocortex and hippocampus were cut using a vibratome (Camden Instruments 7000smz-2) while submerged in aCSF. The brain slices were then placed in an interface holding chamber filled with aCSF used for physiological recordings, which was devoid of kynurenic acid and contained 1.3 mM MgCl_2_, at room temperature for 30 min. The temperature was then slowly increased and maintained at 30 °C. Slices were incubated for at least one hour before being transferred to the recording chamber.

### Pharmacological interventions

Stock solutions were prepared for all compounds in regular aCSF, as all the drugs used are water-soluble. The stocks were diluted into aCSF and perfused. NMDA (30 µM, 10 min) was applied to induce excitotoxic injury in slices, as done previously [71]. We used 4-AP (100 µM, ~ 70 min) to generate seizure-like activity. Hyperosmotic aCSF (+ 40 mOsm) was made by adding 40 mM mannitol to the regular aCSF. All drugs were perfused at ~ 2 mL/min and took less than 30 s to reach the recording chamber.

### Multiphoton imaging

*Acute brain slices (P11–13):* Slices were placed in a submerged chamber that was continuously perfused with aCSF, saturated with carbogen, and maintained at 32–34 °C. The location of the sensory neocortex was determined using epifluorescence. Two-photon laser scanning microscopy (2PLSM) was performed using a Bruker Ultima Galvo-resonant system mounted on an Olympus BX51WIF upright microscope body equipped with a water-immersion objective (20X, 1.0 N.A.). A single Ti: sapphire tunable laser (Mai Tai HPDS; Spectra-Physics) generated two-photon excitation for YFP at 860 nm and for GCaMP6s at 920 nm. Scanning was performed with Galvo mirrors and the Resonant-Galvo system. Emitted light was bandpass filtered at 565 nm using a dichroic mirror (T565lpxrxt, Chroma), and green or yellow emission wavelengths were isolated through a 525/35 nm filter. GaAsP or multi-alkali photomultiplier tubes (PMT, Hamamatsu Photonics) were used to acquire signals simultaneously. Images were acquired in Layer IV/V of the sensory neocortex. Three-dimensional stacks (3D) of raster scans in the XY plane were imaged at 2 μm intervals (z-axis) with a 512 × 512-pixel resolution. Time series acquisition of single XY planar raster scans was performed using resonant-galvo scanning at a 512 × 512-pixel resolution at 29.67 frames per second. Images displaying dendritic beading were acquired using a 2X digital zoom. 3D raster stacks focusing on dendritic spines were acquired at 1024 × 1024-pixel resolution and 8X digital zoom. Planar time series acquisitions focusing on dendritic Ca^2+^ transients were acquired using resonant-galvo scanning at 512 × 512-pixel resolution and 7-8X digital zoom.

### *Electrical stimulation and evoked Ca*^*2*+^*transients*

A stainless steel monopolar stimulating electrode was placed in the deep neocortical layers IV/V. Responses to increasing stimulus duration and intensities were measured before selecting stimulus parameters that evoke a reliable, near-peak response (9 V, 100 µs). Single electrical stimuli were delivered during baseline and 40 min post-NMDA. Time series of evoked responses were recorded for 60 s using resonant-galvo scanning at 29.9 Hz and later down-sampled to 7.48 Hz.

### *Awake, behaving mice (P14*–*17)*

Surgical window implantation for in vivo imaging was performed on the recording day. Anesthesia was induced with 5% isoflurane inhalation using the SomnoSuite delivery system (Kent Scientific Corporation). Mice were mounted on a stereotactic assembly with a nose-cone mask suitable for neonatal mice. A circular metal headplate was mounted on the skull with cyanoacrylate glue and dental cement (Stoelting #51458 & 51456). A circular craniotomy (∼3 mm diameter) was drilled over the sensory neocortex. When removing the skull, measures were taken to minimize damage to the dura and underlying cortex [27]. The brain was hydrated with saline or saline-soaked surgical foam. A perforated cover glass was placed over the open craniotomy and sealed with a barrier made from cyanoacrylate glue and dental cement. The mice were transferred and head-fixed to a treadmill equipped with a rotary encoding system (YUMO E6B2-CWZ3E sensor, Netzor precision). We used a Bruker–Ultima resonant-galvo system with a Nikon LWD water immersion objective (16X, 0.8 N.A.) for in vivo imaging. Images were acquired at depths ranging from 150 to 300 µm below the surface. GCaMP6s and YFP were excited at a wavelength of 920 nm, and emission was filtered through a 525/35 nm filter and acquired using a single GaAsP PMT (Hamamatsu Photonics). Mouse behaviors were recorded using an IR-sensitive CMOS camera (Basler acA4112). Longitudinal time-series acquisition of single XY planar raster scans was performed using resonant-galvo scanning at a 512 × 512-pixel resolution at approximately 29.98 frames per second (2 × digital zoom). Volumetric Z-stacks were acquired using galvo-galvo scanning at a 512 × 512-pixel resolution, approximately 0.87 frames per second, with 2×, 4×, or 8× digital zoom. Motion artifacts in the XY and Z dimensions were minimal, and XY motion artifacts were corrected by a motion correction algorithm (Image-stabilizer, template update coefficient: 0.9, error tolerance: 1*10^–7^, ImageJ).

### Mesoscopic imaging

Slices were placed in a submerged chamber attached to the stage of an upright microscope (Olympus MVX10), continuously perfused with aCSF saturated with carbogen and maintained at 32–34 °C. Excitation was delivered by an LED light (X-Cite XYLIS XT720S). Fluorescent mesoscopic imaging of the entire brain slice was performed using a 2 × objective (NA 0.5). The LED light was band-passed through a 470/40 nm filter and a dichroic 495 nm filter, while the emitted light was isolated using a 525/50 nm filter. Images were acquired with a CMOS camera (Basler Ace acA4112-30 µm) at a frame rate of 10 Hz with 4:1 binning. The camera acquisition and LED excitation were digitally synchronized using Micro-manager (ImageJ).

### Extracellular electrophysiology in acute brain slices

*NMDA perfusion:* Acute brain slices were placed in an interface chamber (32–34 °C) and perfused with aCSF saturated with carbogen. Glass electrodes filled with aCSF were placed in the neocortex (layers IV/V of somatosensory regions), identified using a stereomicroscope (AmScope). Extracellular field potentials were recorded using a low-noise differential amplifier (DP-311, Warner Instruments, 100 × gain) and digitized at 2 kHz (IX/408, iWorx Systems Incorporated). NMDA was applied to the slices (30 µM, 10 min) to induce an acute excitatory condition. Field potentials at baseline, during NMDA treatment, and washout were analyzed using a custom-written macro in Igor Pro 8 (WaveMetrics) to perform a Short-Time Fourier Transform (a Fast-Fourier Transform every 30-s window) [19].

*Hippocampal long-term potentiation*: Transverse (400 µm) sections were prepared from the isolated hippocampi as described previously [1, 50]. Slices were quickly transferred onto a net insert in an interface recording chamber (Fine Science Tools, Foster City, CA) and perfused with aCSF (1 mL/min at 32–34 °C) in a humidified carbogen atmosphere for at least 2–3 h before the recordings began. Field potentials were recorded in CA1 *stratum radiatum* by stimulating Schaffer collaterals with a monopolar electrode (#571000, A-M Systems), and recordings were obtained using aCSF-filled glass microelectrodes (2–5 MΩ resistance). Biphasic stimuli were delivered (100 μs per phase) every minute using an intensity that evoked ~ 50% of the maximal response determined by an input–output curve for each experiment. A stable baseline was recorded for at least 20 min before NMDA application and the induction of a subsequent long-term potentiation by four massed trains of 100 stimuli (100 Hz for 1 s), with an inter-train interval of 5 s.

### Data analysis

*Dendritic beading quantification*: The 2PLSM 3D raster stacks from Thy1-YFP-H slices were background subtracted and filtered (median: 2) before generating maximum intensity projections (MIPs). Segmented line ROIs were drawn to trace individual dendritic branches. Dendritic ROIs were matched at various time points using a custom-built macro (ImageJ). Linear profile plots or traces of dendritic YFP signals were generated (ImageJ). YFP fluorescence values across linear dendritic ROIs were median-filtered (medfilt1), and beads were identified as the peaks (FindPeaks) in the YFP signal along the length of the dendrite (MATLAB R2019a, MathWorks). To detect dendritic beads using Thy1-GCaMP6s slices, the 3D raster stacks were background-subtracted, smoothed (Kuwahara, sampling window = 5), and split into maximum intensity projections (MIPs) for every 10 images (20 μm depth). A threshold (5*SD grayscale) was used to apply a binary mask over MIPs, and dendritic beads were segmented using the “analyze particles” function (ImageJ, size 2–50 μm, circularity: 0.75–1). To eliminate false detections, all detected beads were manually checked for accuracy (false detection rate: 7.8 ± 3.7%).

*Sholl analysis:* 90–120 µm thick 2PLSM 3D raster stacks of neuronal somas were acquired 40 min after NMDA application. The stacks were background-subtracted, filtered, and MIPs were generated. The MIPs were converted to binary using a threshold intensity (mean + 3*SD). Using a custom-built macro (ImageJ), concentric circles (20 µm radius increments) were drawn from the ROI centroid of the neuronal cell body. Dendritic beads within each 20 µm disc were identified and counted using the analyze particle function (ImageJ, size 2–50 μm, circularity: 0.75–1).

*Dendritic spine quantification:* 2PLSM 3D raster stacks (1024 × 1024 pixels, 8X) focusing on dendritic segments with visible spines were acquired at baseline and 40 min after NMDA application. The stacks were background-subtracted, smoothed using a linear Kuwahara filter (sampling window = 5), and converted to binary MIPs (threshold: mean + 3*SD). The binary images were skeletonized to visualize the spines connected to the dendritic branches (ImageJ). Segmented line ROIs were drawn to trace individual dendritic branches. Spines connected to each branch were manually identified and counted using the skeletonized MIP and Z-stacks. Spines that did not show connections to dendritic branches after thresholding and skeletonization were excluded from the paired analysis.

*Dendritic Ca*^*2*+^ *transient measurements:* Raw images in time series were background-subtracted, smoothed (median filter, radius = 2), and compressed into MIPs. Dendritic Ca^2+^ transients were isolated using multi-point or linear ROIs drawn across the length of the dendritic branch (ImageJ). Fluorescence signal traces were collected from each ROI (ImageJ). Fluorescence traces were analyzed in MATLAB R2019a (MathWorks) and Prism 9 (GraphPad). To determine the characteristics of evoked Ca^2+^ responses, we performed baseline subtraction and normalization (ΔF/F_0_) of the neuropil-subtracted Ca^2+^ signal for each neuron employing the following equation: (Ft-F_0_)/F_0_, where Ft is the fluorescence intensity of a given frame, and F_0_ is the average fluorescence of the baseline (first 8–10 s of the trial before stimulation). Ca^2+^ response parameters (peak amplitude, locations, width, prominence) were determined using the FindPeaks function in MATLAB (minimum peak prominence: 0.025). Area under the curve (AUC) was then calculated using trapezoidal numerical integration (MATLAB, Trapz function) [71].

### Statistical analysis

Experimenters were not blinded during data analysis, as the neuronal Ca^2+^ activity and dendritic beading elicited by excitotoxic insults were apparent. The normality of distributions was assessed using the D’Agostino–Pearson K2, Anderson–Darling tests, and QQ plots. Data are presented as mean ± confidence interval (95% CI) or median ± interquartile range (IQR) based on the normality of the distribution. Outliers were detected using Grub’s or IQR tests (for parametric and nonparametric data), although no outliers were detected or excluded. Matched data representing multiple time points with the same perturbation were analyzed using repeated measures One-way ANOVA (or mixed-effect model if data were missing) with Dunnett’s test for multiple comparisons (Friedman’s test with Dunn’s multiple comparison tests for non-parametric data). Unpaired parametric data at various time points were analyzed using One-way ANOVA (or Kruskal–Wallis test for non-parametric data). Geisser–Greenhouse correction was used whenever appropriate. Data with multiple time points were compared across groups using Two-way ANOVA with Tukey’s or Sidak’s test for multiple comparisons. Data points representing individual slices/cells were compared across groups using a two-tailed, unpaired t-test (parametric data) or Mann–Whitney U test (non-parametric data). Spearman’s rank correlation (ρ) and Pearson’s correlation (expressed as r) were used for non-parametric and parametric data, respectively. Paired comparisons were performed using a two-tailed, paired t-test. Normalized distributions of individual events were compared across genotypes using a two-sample Kolmogorov–Smirnov (KS) test. Statistical significance was considered at p < 0.05.

## Results

### Excitotoxicity induces dendritic beading in the neocortex and hippocampus during early development.

**Fig. 1:**
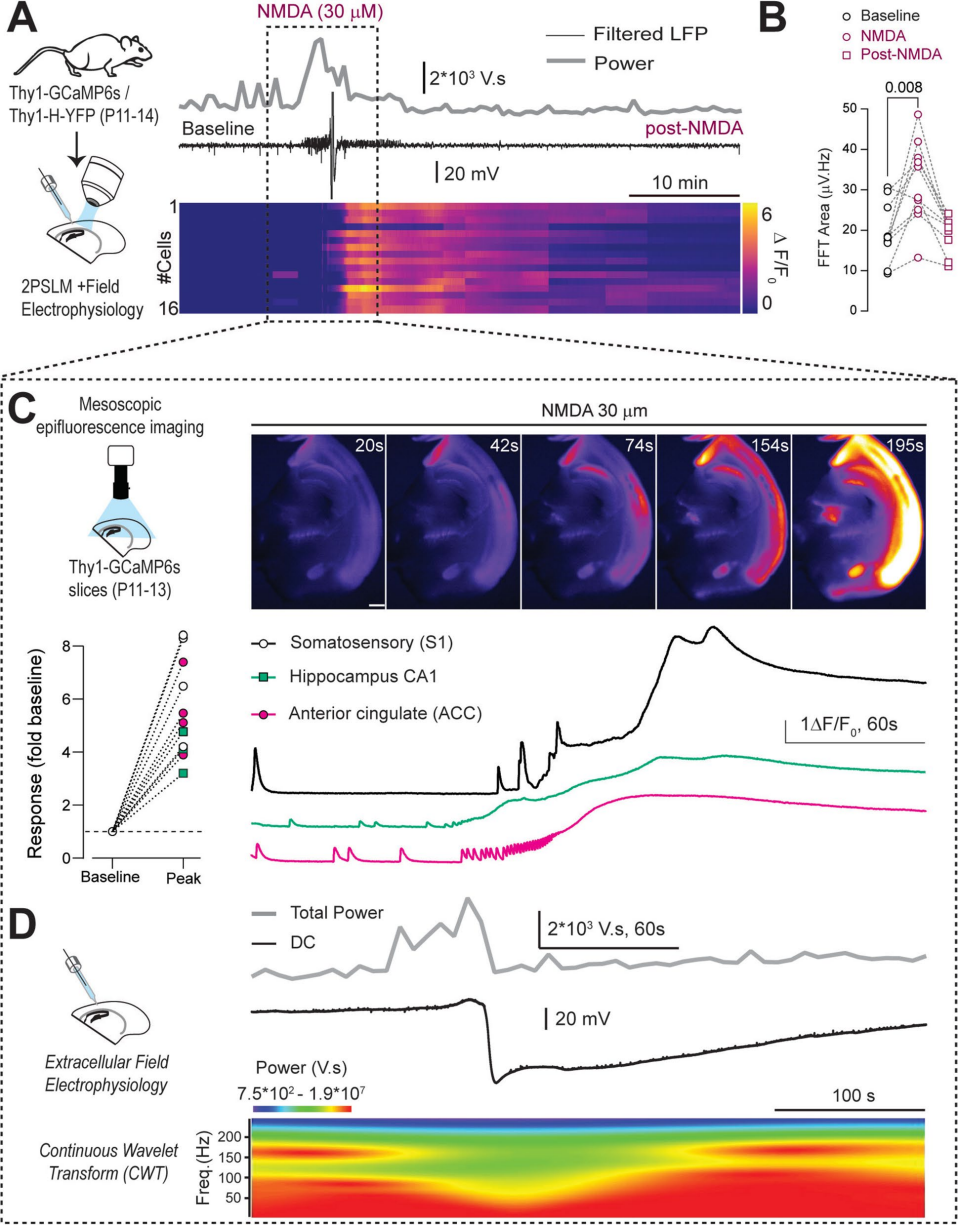
Brief NMDA perfusion induces synchronous activity followed by spreading depolarization. **A**, Left: Experimental model and approach. Right: Representative trace showing filtered (1 kHz, low-pass) local field potentials (LFP) and its Short-Time Fast-Fourier Transform (FFT) power calculated using a 30-s window, along with a heatmap of Ca^2+^ transients derived from neocortical neuronal ROIs (P11–13, Thy1-GCaMP6s slices). **B**, FFT area from individual neonatal slices at baseline, during NMDA application, and post-NMDA (RM One-way ANOVA with Geisser–Greenhouse correction, F(1.24, 11.2) = 16.3, p = 0.0013, n = 10 slices). **C**, Top: Experimental design and images showing the propagation of Ca^2+^ transients across the entire brain slice, captured using mesoscopic fluorescent imaging. Bottom: Peak Ca^2+^ transient quantification elicited during NMDA application in different brain regions (somatosensory, anterior cingulate cortices, and hippocampal CA1, n = 4 slices) and example traces of synchronous Ca^2+^ activity. **D**, Representative trace showing FFT power, direct current (DC) signal, and a spectrogram showing power spectral densities (0–300 Hz, 30 s bins) during NMDA perfusion. Fast-Fourier transform of LFP, followed by continuous wavelet transformation and spectrogram, was performed using Igor Pro 8. Scale bar: 1 mm. See also Video S1

**Fig. 2:**
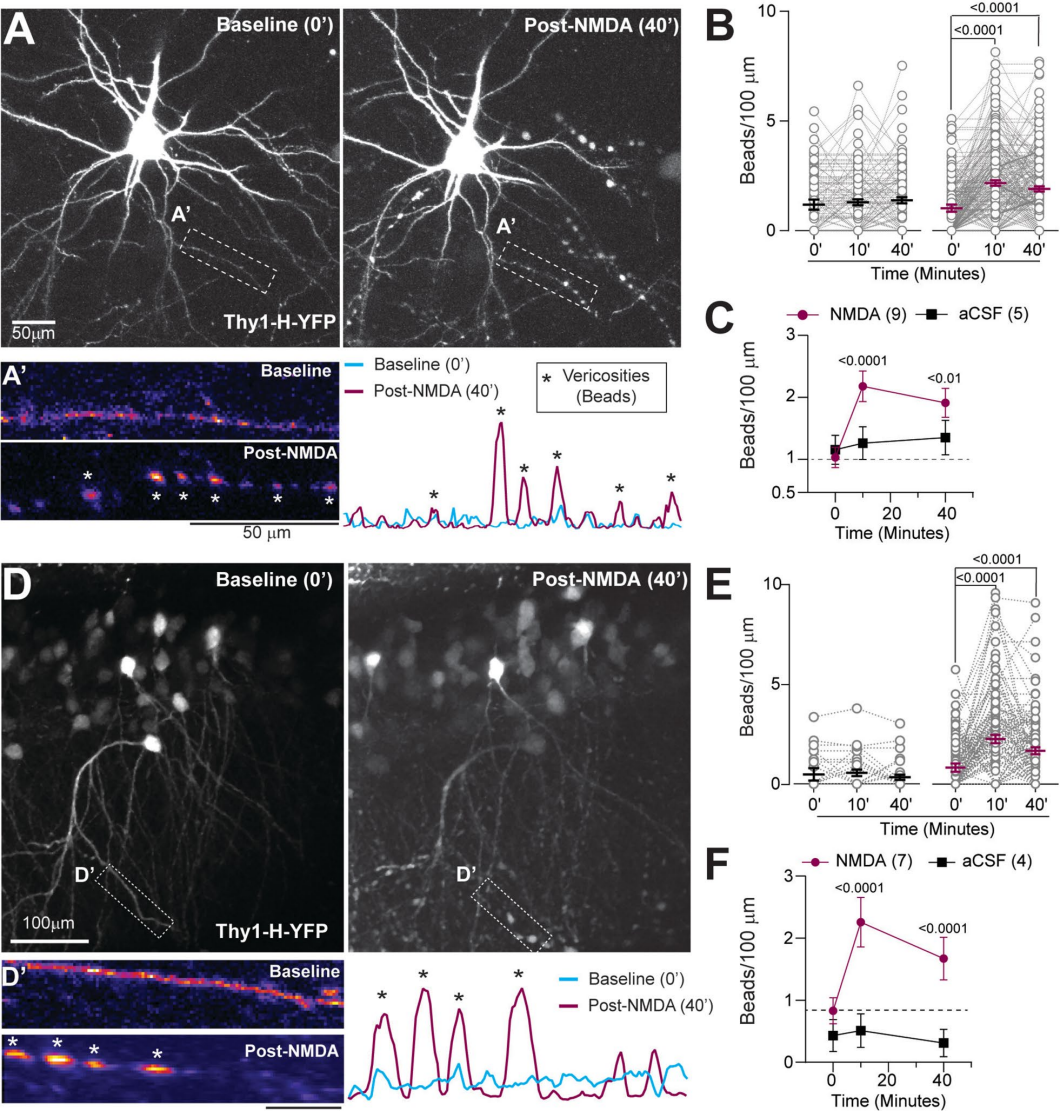
NMDAR stimulation induces dendritic beading during early brain development. **A**, Maximum intensity projections (MIPs) were generated using Z-stacks of neocortical neurons (P11–13) during baseline (0′) and post-NMDA treatment (40′). **A**′, Example of a dendrite at 0′ and 40′. Varicose beading was quantified as punctate in the YFP signal (*, peak signals in linear profile plots). **B**, NMDA induced significant dendritic beading at 0′ and 40′ (RM One-way ANOVA with Dunnett’s tests. aCSF: F(1.9, 212) = 1.17, p = 0.31, n = 3:5:114 (mice: slices: dendrites), NMDA: F(2, 404) = 40.5, p < 0.0001, n = 5:9:203). **C**, NMDA-induced dendritic beading was higher and persistent compared to aCSF (Two-way ANOVA, Interaction: F(4, 1245) = 5.45, p = 0.0002, followed by Tukey’s test). **D**, Like A, but in the dorsal CA1 region of the hippocampus (P11–13). Quantification of beading (D′) using YFP signal localization. **E–F**, Significant and persistent dendritic beading occurred after NMDA application in CA1 neurons (**E**, RM One-way ANOVA with Dunnett’s test. aCSF: F(2, 62) = 1.65, p = 0.2, n = 3:4:53; NMDA: F(1.98, 251) = 24.4, p < 0.0001, n = 4:7:128; **F**, Two-way ANOVA, Interaction: F(2, 486) = 4.72, p = 0.009, followed by Tukey’s test. Data: Mean ± CI

We studied the functional consequences of dendritic beading induced by NMDA in acute brain slices during early brain development (P11–13). We chose this model because brief NMDA exposure mimics intense seizure-like events, which terminate in spreading depolarizations [20, 25, 65, 76] and induce robust dendritic beading in neuronal cultures, adult acute brain slices, and in vivo [28, 30, 46]. First, we evaluated whether we could observe similar findings in the developing brain. Using extracellular field electrophysiology and mesoscopic imaging in acute brain slices prepared from Thy1GCaMP6s pups (P11–13), we found that brief NMDA treatment (30 μM for 10 min) induced synchronous field potential activity resulting in increased Short-Time Fast-Fourier Transform (FFT) power, followed by a prolonged depolarization causing a DC shift and massive neuronal Ca^2+^ influx (Fig. 1, Video S1). To evaluate dendritic beading, we repeated this experiment using acute brain slices from Thy1-YFP pups, which have a sparse neuronal YFP expression during early development. A few dendrites showed beads at baseline (Fig. 2 and additional file, Fig. S1). NMDA perfusion caused a robust and persistent dendritic beading in neocortical neurons, which lasted up to 40 min of washout (Fig. 2A–C). As in the neocortical neurons, brief NMDA receptor (NMDAR) stimulation also induced robust dendritic beading in the dorsal CA1 region of the hippocampus (Fig. 2D–F).

**Fig. 3:**
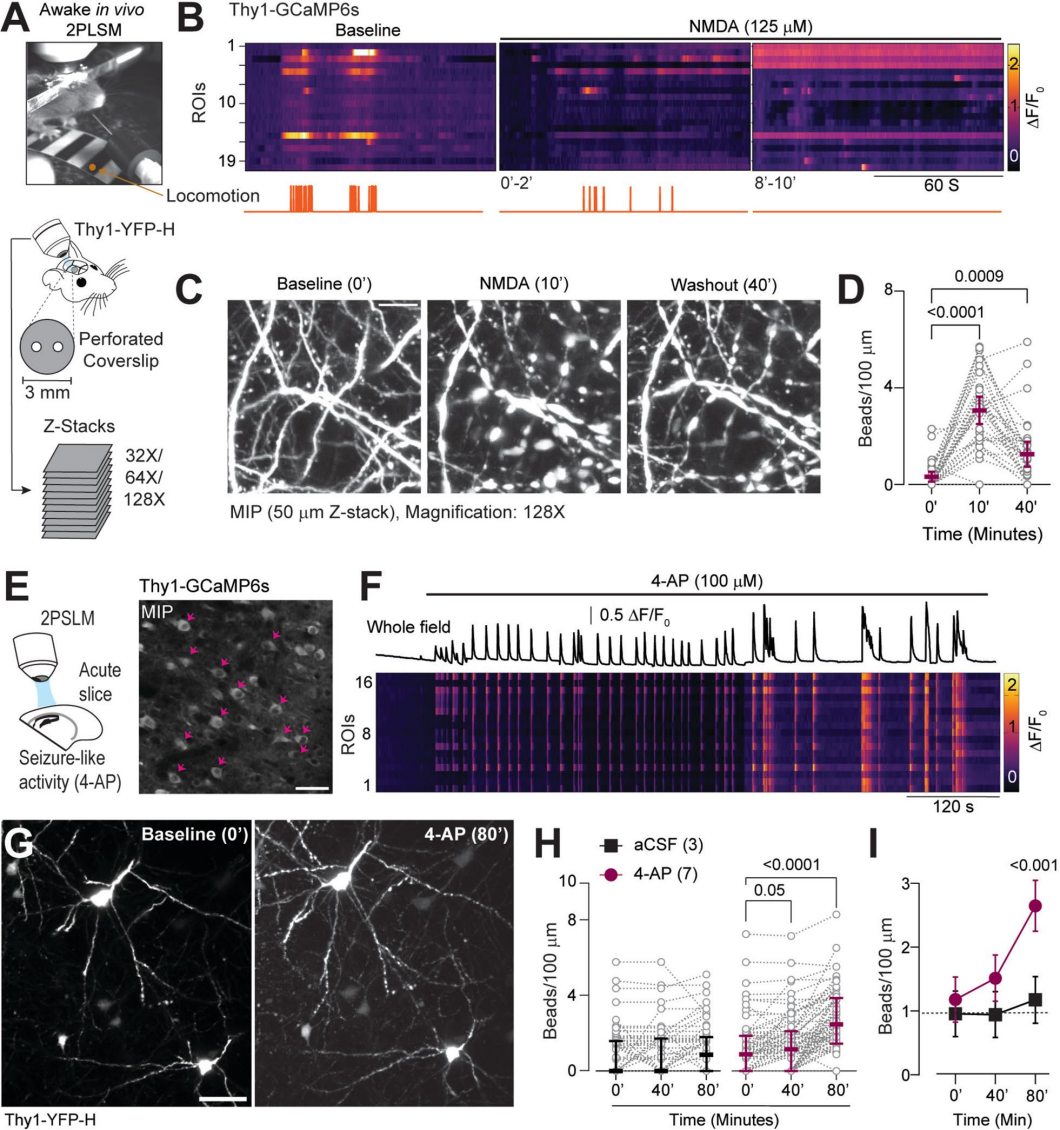
NMDA application in vivo and recurrent seizures in acute brain slices result in dendritic beading. **A**, Experimental approach for 2P volume stack acquisition in awake behaving mice during early development (P14–17). **B**, Heat map raster showing representative (somatic) Ca^2+^ transients from neocortical neurons in an awake, behaving mouse pup (Thy1GCaMP6s, P16), along with a binary trace of corresponding mouse locomotion. **C**, Max intensity projection (MIPs) of neuronal dendrites expressing YFP (Thy1-YFP-H) acquired during baseline (0′), NMDA treatment (10′), and washout (40′). **D**, Significant increase in the bead densities in vivo at 10′ and 40′, compared to baseline (Friedman test, p < 0.0001, followed by Dunn’s test, n = 3:34 (mice: dendrites)). **E**, Left: Experimental model and approach. 2PLSM was performed on acute brain slices at baseline and during 4-AP (100 µM, up to 90′, see materials and methods). Right: Example MIPs (Thy1-GCaMP6s) showing selected neocortical neurons (arrows) with Ca^2+^ transients shown in panel F. **F**, Representative trace of whole-field and heatmap of neuronal Ca^2+^ transients during prolonged 4-AP application. **G**, MIPs (Thy1-YFP-H) at baseline (0′) and after protracted 4-AP treatment (80′). **H–I**, Prolonged 4-AP application induced dendritic beading, especially at 80′ (**H**, Friedman with Dunn’s post hoc tests. aCSF: p = 0.038, but not significant between groups (p > 0.05); 4-AP: p < 0.0001; **I**, Two-way ANOVA with Sidak’s test, Interaction: F(2, 378) = 6, p = 0.003); Treatment: F(1, 378) = 24.7, p < 0.0001). Scale bars: 50 µm. Data: Mean ± CI (Median ± IQR for **D** and **H**). See also Video S2

We then evaluated whether brief NMDAR stimulation also induces dendritic beading in awake, behaving mice during early development. Cranial windows with perforated cover glass were implanted on young Thy1-GCaMP6s and Thy1-YFP-H mice (P14–17) and were head-fixed on a rotary-encoding-capable treadmill (Fig. 3A). We used slightly older mice, as it is challenging to implant cranial windows in neonatal pups when the skull is not fully calcified [42, 82]. In Thy1-GCaMP6s mice, neuronal Ca^2+^ transients coincided with locomotion at baseline. NMDA (125 µM, 10 min) was applied through the perforated cover glass, resulting in a robust increase in neuronal Ca^2+^ activity and a complete locomotion arrest at 10 min (Fig. 3B, Video S2). In awake, behaving Thy1-YFP-H mice, brief NMDAR stimulation resulted in significant beading of the dendrites that lasted for at least 40 min, similar to the findings in acute brain slices (Fig. 3C–D). Despite the difference in ages, the change in dendritic bead densities between baseline and post-NMDA timepoints was not different between P11–13 and P14–17 pups (Δ beads/100 µm, 40′–0′; P11–13: mean = 0.85, SD = 1.87, n = 9 slices (211 dendrites); P14–17: mean = 0.89, SD = 1.34, n = 3 mice (34 dendrites); p = 0.88, t-test with Welch’s correction).

As robust NMDA receptor activation occurs during recurrent refractory seizures [34, 86], we investigated whether recurrent seizures in developing brain circuits (P11–13) also cause dendritic beading. We used 4-aminopyridine (4-AP), a chemoconvulsant commonly used to induce seizure-like activity in vitro, which we also have employed previously [7, 19, 44, 55]. Perfusion of 4-AP resulted in synchronous neuronal Ca^2+^ spikes that were significantly more frequent and prominent compared to spontaneous synchronous activity at baseline (Additional file, Fig. S2). Afterwards, the 4-AP-induced synchronous neuronal Ca^2+^ spikes became less frequent and longer in duration over time (Fig. 3E, F). In Thy1-YFP-H acute brain slices, prolonged 4-AP application induced dendritic beading in developing neocortical neurons at 40 min, with a significant increase after 80 min (Fig. 3G–I), similar to NMDA perfusion. These results demonstrate that excitotoxic injury, whether by NMDA or seizure-like activity, leads to dendritic beading in the early developing brain.

### Excitotoxic injury leads to a uniform spatial distribution of dendritic beads and the loss of spines in the developing brain.

**Fig. 4:**
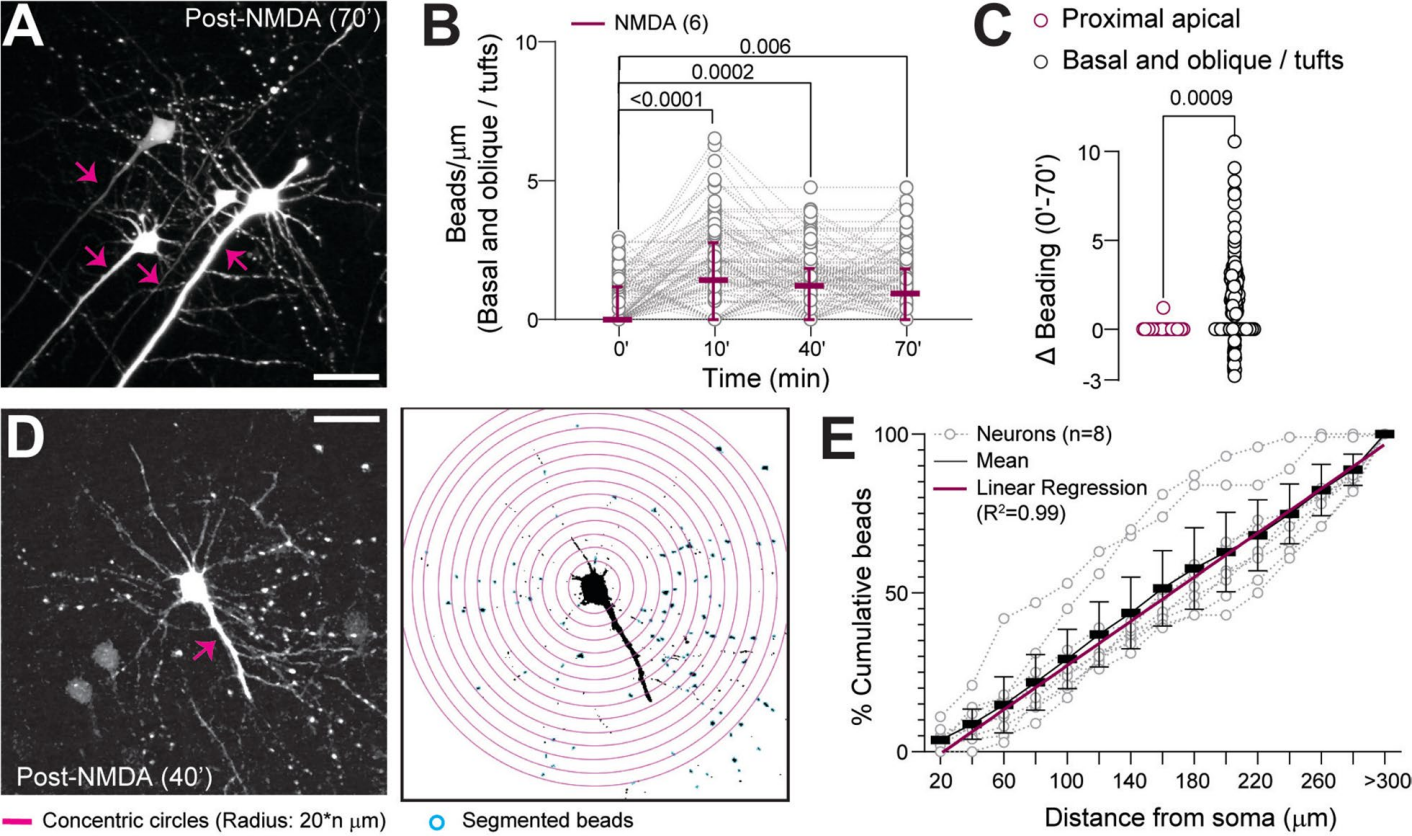
Uniform spatial distribution of beads in the basal and oblique dendritic segments after NMDA. **A**, Example MIP showing robust, long-lasting (up to 70′ post-NMDA) beading in basal dendrites and oblique branches/tufts of apical dendrites, with no beading in the principal branches of apical dendrites proximal to neuronal cell bodies (arrows). **B**, Quantification of increased bead density up to 70′ post-NMDA (Friedman test, p < 0.0001, Dunn’s post-hoc test). **C**, Difference in NMDA-induced dendritic beading in proximal apical dendrites and other dendritic segments (Mann–Whitney test, u = 2459, p = 0.0009, n = 12: 23: 349 (slices: neurons: dendrites)). **D**, Left: Example MIP of a neocortical neuron with beaded dendrites (post-NMDA treatment at 40′). Right: Sholl analysis using concentric circles at 20 μm intervals, starting from the soma, up to 300 μm radius. **E**, Quantification of the spatial location of dendritic beads by Sholl analysis (number of dendritic beads within the concentric circles). Linear regression, R^2^ = 0.998. Scale bars: 50 μm. Data: Mean ± CI and median ± IQR for C, n = 3:8:8 (mice: slices: neurons)

Studies in adult mouse brain slices indicate that excitotoxicity-induced beading first appears in distal dendrites and then progressively develops in proximal segments [28, 29]. Progressive beading of proximal apical dendrites is often considered a harbinger of neuronal injury, which can lead to cell death [45, 74]. We hypothesized that a similar distal-to-proximal beading direction would also occur after excitotoxicity during early brain development. Following brief NMDAR stimulation, we observed robust dendritic beading in basal dendrites and oblique branches of the apical dendrites in Thy-YFP-H acute brain slices (Fig. 4A, B). However, unlike studies in the adult brain, the proximal segments of the apical dendrites of neonatal neurons did not show progressive beading, even 70 min following NMDA (Fig. 4C). The Sholl analysis demonstrated a uniform distribution of beads in 3D space away from the soma (Fig. 4D), resulting in a linear relationship between the cumulative number of beads and distance (Fig. 4E). Therefore, in the developing brain, unlike in adults, the apical dendrite proximal to the soma do not exhibit progressive beading over time.

**Fig. 5:**
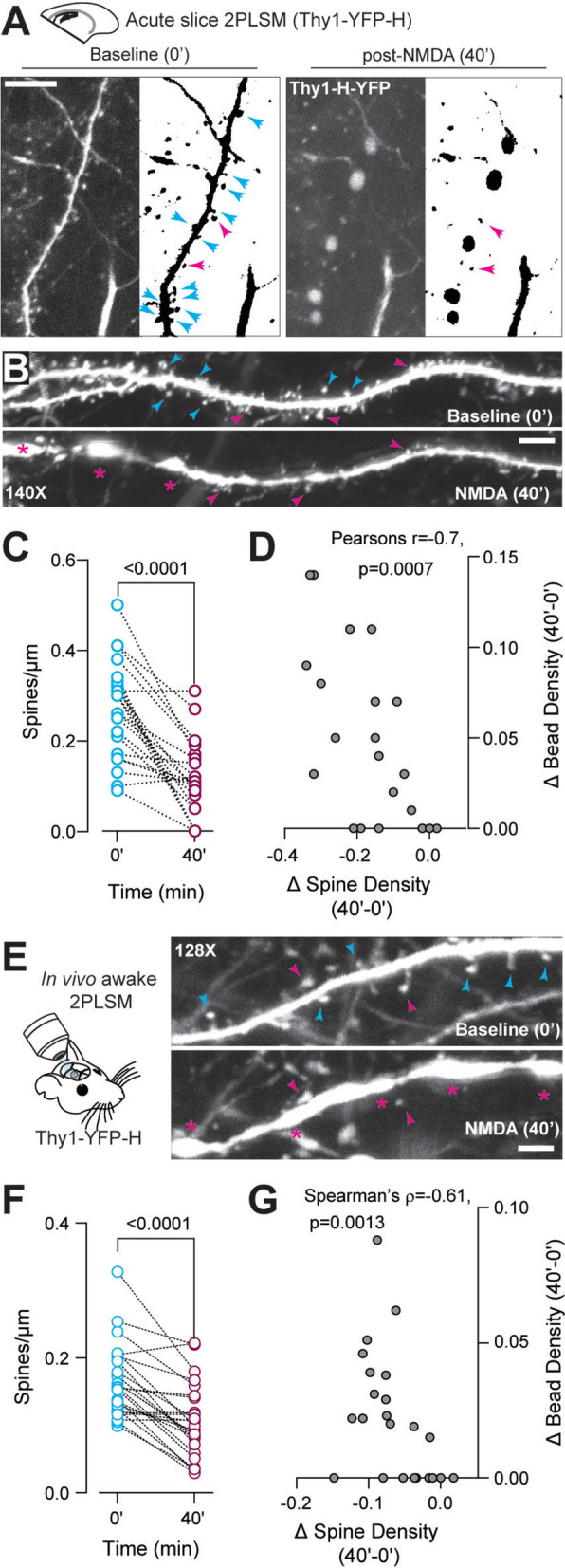
Decrease in spine density by NMDA-induced dendritic beading ex vivo and in vivo. **A**, Example MIPs represent Z stacks of YFP-expressing dendrites, at high magnification (140x) and resolution (1024 × 1024). The MIPs were converted to binary and skeletonized to identify dendritic spines connected to specific dendritic branches (right). Arrows depict examples of post-NMDA spine loss and stable spines (cyan and magenta, respectively). **B**, Example MIPs of a single dendritic segment at 0′ and 40′, showing localized beads (*) at one end and some intact spines at the opposite end. A subset of lost and stable spines is indicated with cyan and magenta arrows, respectively. **C**, NMDA induced significant loss of dendritic spines post-NMDA treatment (at 40′, two-tailed paired t-test, p < 0.0001, n = 30 paired dendrites, 5 slices, 3 mice). **D**, The extent of dendritic spine loss correlated with the degree of dendritic beading (Pearson's r = − 0.7, p < 0.0007). **E**, In vivo experimental design (left) and example MIPs of a single dendritic segment at 0′ and 40′ acquired at high magnification (128x). Beads, stable spines, and lost spines are depicted as asterisks, magenta, and cyan arrows, respectively. **F**, Significant loss of spine density at 40′ post-NMDA in vivo (two-tailed paired t-test, p < 0.0001, n = 25 paired dendrites from 3 mice). **G**, The extent of dendritic spine loss correlated with the degree of dendritic beading in vivo (Spearman ρ = − 0.61, p = 0.0013). Scale bars: 10 μm for **A**, 2 μm for **B**, **E**

Data from mature neurons and adult human epilepsy patients suggest that dendritic beading is associated with significant spine loss [12, 72]. We next evaluated the effect of dendritic beading on neuronal spine densities in the developing neocortex. First, we measured spine densities in multiple dendritic segments in acute brain slices using 3D 2PLSM raster stacks (1024 × 1024, 140 ×) at baseline and 40 min after brief NMDAR stimulation. Dendritic beading and spine densities were quantified from the same dendritic segments during baseline and post-NMDA treatment (Fig. 5A). Spine loss was not universal, as 16.67% of the analyzed dendrites did not show spine loss after NMDA. In some dendrites, beading and consequent spine loss were localized to specific segments. Interestingly, dendritic regions lacking beads exhibited intact spines (Fig. 5B). Although not ubiquitous, we found that brief NMDAR stimulation induced a significant loss of dendritic spines (Fig. 5C). The extent of dendritic spine loss was correlated with dendritic bead density after NMDA (Fig. 5D). We then investigated spine loss after brief NMDAR stimulation in awake behaving mice, using 3D 2PLSM raster stacks (1024 × 1024, 64–128×, Fig. 5E). Similar to acute brain slices, we found that brief NMDAR stimulation in awake behaving mice resulted in a robust reduction in spine densities (Fig. 5F), and the decrease in spine densities was correlated with an increase in dendritic beads (Fig. 5G). These data indicate a strong relationship between dendritic bead formation and spine loss.

### *Dendritic beads demonstrate a sustained increase in Ca*^*2*+^*and impede signal propagation.*

**Fig. 6:**
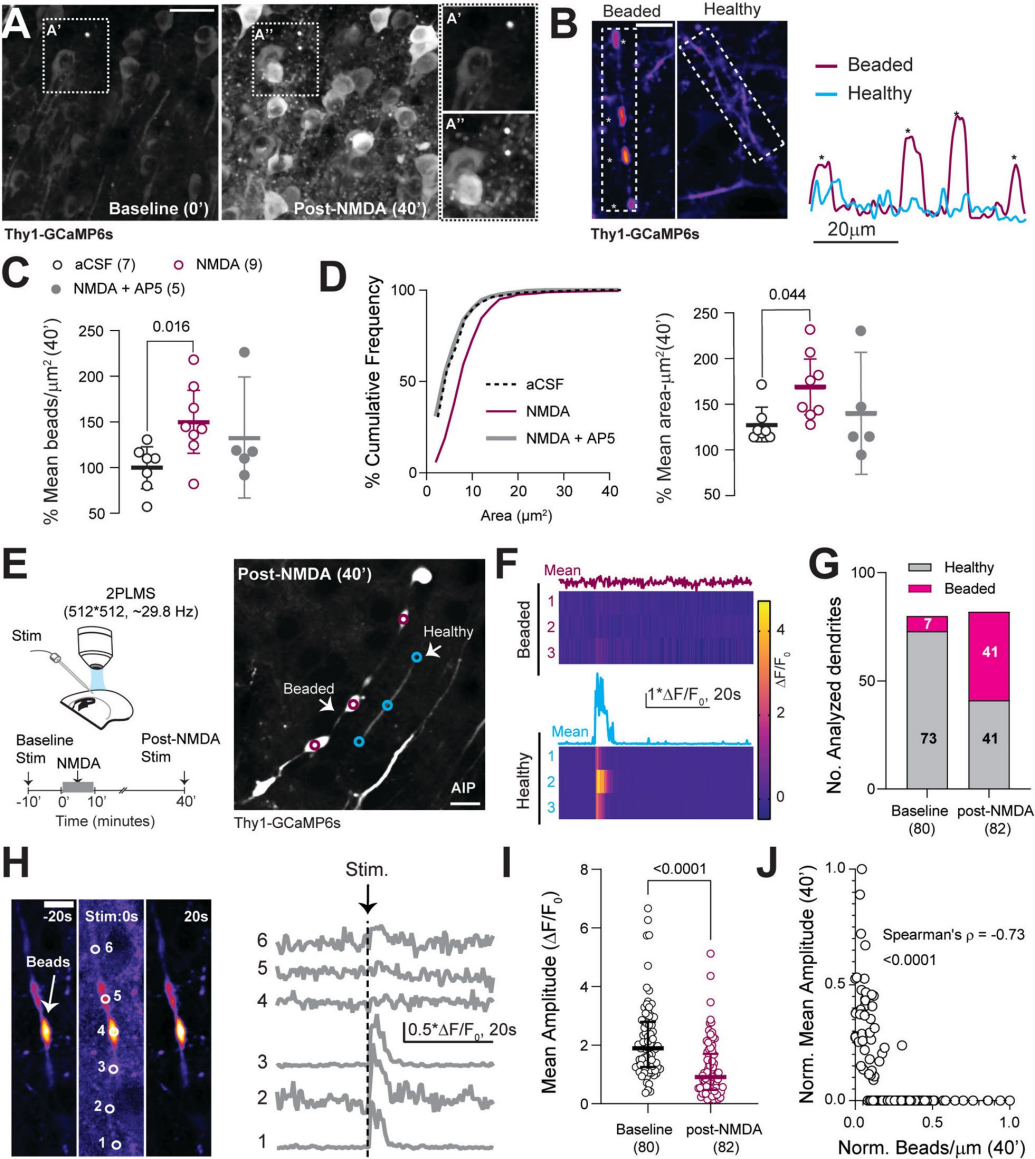
Dendritic beads have a persistent Ca^2+^ elevation and hinder the propagation of Ca^2+^ transients. **A**, Example images of Thy1-GCaMP6s slices at baseline (0′) and post-NMDA treatment (40′). Inset: NMDA-induced increase in punctate GCaMP6s signal within the neuropil regions, representing beads with elevated Ca^2+^. **B**, MIPs of beaded and healthy dendritic segments captured at high magnification and spatial resolution (120x, 1024 × 1024 resolution, Scale: 10 μm) post-NMDA treatment (40′). Plot profiles (raw grayscale values) of linear dendritic ROIs show a high contrast in the resting Ca^2+^ signal between dendritic beads and intact dendrites. This high contrast in the resting Ca^2+^ was used to identify dendritic beads in slice preparations from Thy1-GCaMP6s pups. **C**, NMDA induced an increase in bead density (One-way ANOVA, F(2,17) = 9.28, p = 0.0019, Tukey’s post-hoc test), which was reversed by AP5. **D**, Frequency distribution of dendritic areas in aCSF, NMDA, and NMDA + AP5 conditions and mean bead area/µm^2^ (p = 0.0036, Kruskal–Wallis test with Dunn’s post-hoc test). **E**, Experimental design (left). Average Intensity Projection (AIP, right) of time-series capturing stimulus-evoked Ca^2+^ transients in healthy and beaded dendrites. **F**, Ca^2+^ activity from ROIs placed along the length of beaded and healthy dendrites and their respective means. **G**, Increase in the proportion of beaded dendrites post-NMDA treatment (Chi-square test, df (33.05, 1), p < 0.0001, n = 80/82 (aCSF/post-NMDA) dendrites, 6 slices, 3 mice). **H**, Representative images of a beaded dendrite post-NMDA application (P12, > 40′) before, during, and after stimulation (left). Stimulus-evoked Ca^2+^ transients from 6 different ROIs throughout the length of the dendrite (right). Propagation of the Ca^2+^ transient stops at the region with dendritic beads (ROI: 4, 5). **I**, Reduction in the mean amplitudes of dendritic Ca^2+^ transients 40′ post-NMDA treatment (Mann–Whitney test, U = 1539, p < 0.0001). **J**, Dendritic Ca^2+^ response mean amplitudes were inversely correlated to the bead density (Spearman’s ρ = − 0.73, p < 0.0001). Scale bars: 50 µm for **A**, **B**; 10 µm for **E**, **H**. Data: Mean ± 95%CI (**C**) or median ± IQR (**D**, **I**). See also Video S3

In addition to the morphological changes associated with excitotoxicity-induced dendritic beading, we also evaluated its physiological consequences using Thy1-GCaMP6s mice, which have sparse neuronal expression of this Ca^2+^-sensitive fluorophore. After NMDA perfusion, and consistent with our previous findings [71], we observed persistently elevated GCaMP6s signal, indicating elevated cytosolic Ca^2+^ (Fig. 6A). Similar to the elevated cytosolic Ca^2+^, we found a subset of affected dendrites with persistently elevated Ca^2+^ signals within the beads, which were discernible from the “healthy” dendrites with no beading (Fig. 6B). Using the differences in Ca^2+^ signals between dendritic beads and healthy dendrites along with image smoothing (Additional file, Fig. S3), we implemented an automated segmentation to identify dendritic beads in Thy1GCaMP6s slices. We observed NMDA-induced increase in the bead densities and their areas, which was prevented by pretreating slices with 50 µM of AP5, an NMDAR antagonist (Fig. 6C, D).

Next, we evaluated the effect of aberrant Ca^2+^ dynamics within the dendritic beads on synaptically-evoked signal propagation. Evoked dendritic Ca^2+^ transients were recorded in Thy1-GCaMP6 slices during baseline and 40 min after inducing dendritic beads with NMDA (Fig. 6E, F). We observed an increase in the proportion of dendrites exhibiting beading following NMDAR stimulation, affecting approximately 50% (41 out of 82) of dendrites compared to around 9.5% at baseline (Fig. 6G). The beaded dendrites exhibited Ca^2+^ transient propagation through the “healthy” regions. However, Ca^2+^ transients were absent in the beaded areas, arresting their propagation (Fig. 6H, Video S3). As a result, the mean amplitude of evoked dendritic Ca^2+^ transients isolated from several ROIs across the dendritic lengths was significantly reduced post-NMDA treatment (Fig. 6I). Furthermore, the average dendritic Ca^2+^ amplitude responses showed an inverse correlation with the bead density (Fig. 6J). These data strongly suggest that excitotoxic dendritic beading impedes signal transmission in the developing brain.

### Excitotoxic dendritic beading reduces hippocampal synaptic plasticity in the developing brain.

**Fig. 7:**
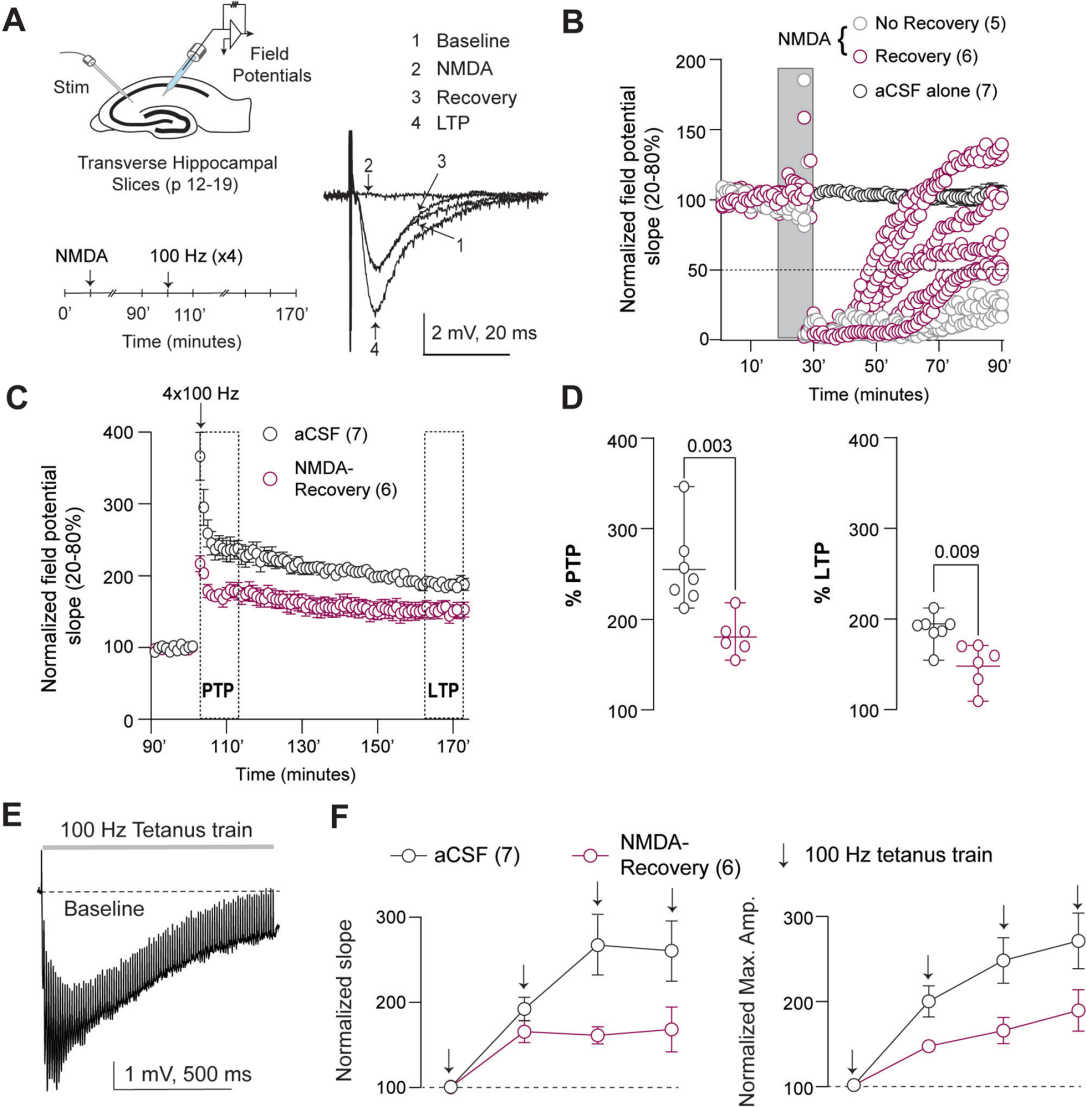
NMDA-induced dendritic beading reduces hippocampal long-term potentiation. **A**, Experimental design (left) and example traces of evoked field potentials (right) at baseline, during NMDA perfusion, recovery, and after long-term potentiation (LTP). **B**, Hippocampal (CA3 to CA1) evoked field potentials were immediately suppressed following NMDA treatment, with only 6 out of 11 hippocampal slices (6 mice) showing recovery (more than 50% of the baseline) after 90 min. **C**, Evoked field potentials at baseline and after induction of LTP using four massed trains (4-MT) of tetanic stimuli in the recovered NMDA-treated slices (Two-way ANOVA, Interaction: F(81,891) = 4.72, p < 0.0001). **D**, Reduction in post-tetanic and LTP compared to controls (% PTP p = 0.003; LTP p = 0.009; unpaired t-test). **E**, Evoked field potentials and baseline drift during the administration of a tetanic train. Data: Mean ± CI. **F**, Gradual potentiation of both normalized slope (20–80%) and maximum amplitude of the first evoked field response following each tetanus train was significantly reduced in the recovered NMDA-treated slices (Two-way ANOVAs, Slope: treatment: F(1, 9) = 5.5, p = 0.044, Amplitude: treatment: F(1, 9) = 5.3, p = 0.047, Mean ± SEM)

Thus far, our results suggest robust, uniformly distributed dendritic beading following brief NMDAR stimulation and prolonged seizures, resulting in diminished and halted dendritic Ca^+2^ transients in the developing brain. Next, we evaluated the impact of excitotoxic dendritic beading on hippocampal plasticity. Hippocampal plasticity, particularly long-term potentiation (LTP) of CA3-CA1 synapses, is recognized as the physiological correlate of learning and memory [8, 9]. We assessed the effect of dendritic beading on hippocampal CA3-CA1 LTP using transverse hippocampal slices prepared from mouse pups at slightly older ages (P14–17, Fig. 7A), since LTP induction is deficient in very young neonatal hippocampal slices [43]. Consistent with suppressed dendritic Ca^2+^ transients, hippocampal (CA3 to CA1) evoked field potentials were temporarily suppressed immediately following NMDA treatment. However, we found that 6 out of 11 hippocampal slices recovered to above 50% of their baseline 70 min post-NMDA (Fig. 7B). We then applied LTP-inducing stimuli (4-massed tetanic trains) [1, 2, 83] to the 6 slices that had recovered responses. After tetanic stimulation, the recovered NMDA-treated slices had a reduction in post-tetanic (0′–10′) and long-term potentiation (60′–70′) compared to controls (Fig. 7C, D). In addition, the gradual potentiation of the field potential responses immediately following the stimulus trains was significantly reduced in the recovered NMDA-treated slices (Fig. 7E, F). These data suggest a strong association between dendritic beading and impaired hippocampal long-term potentiation, a physiological correlate of learning and memory, even in “recovered” slices.

### *Hyperosmotic interventions resolve dendritic beads and restore dendritic Ca*^*2*+^*activity.*

**Fig. 8:**
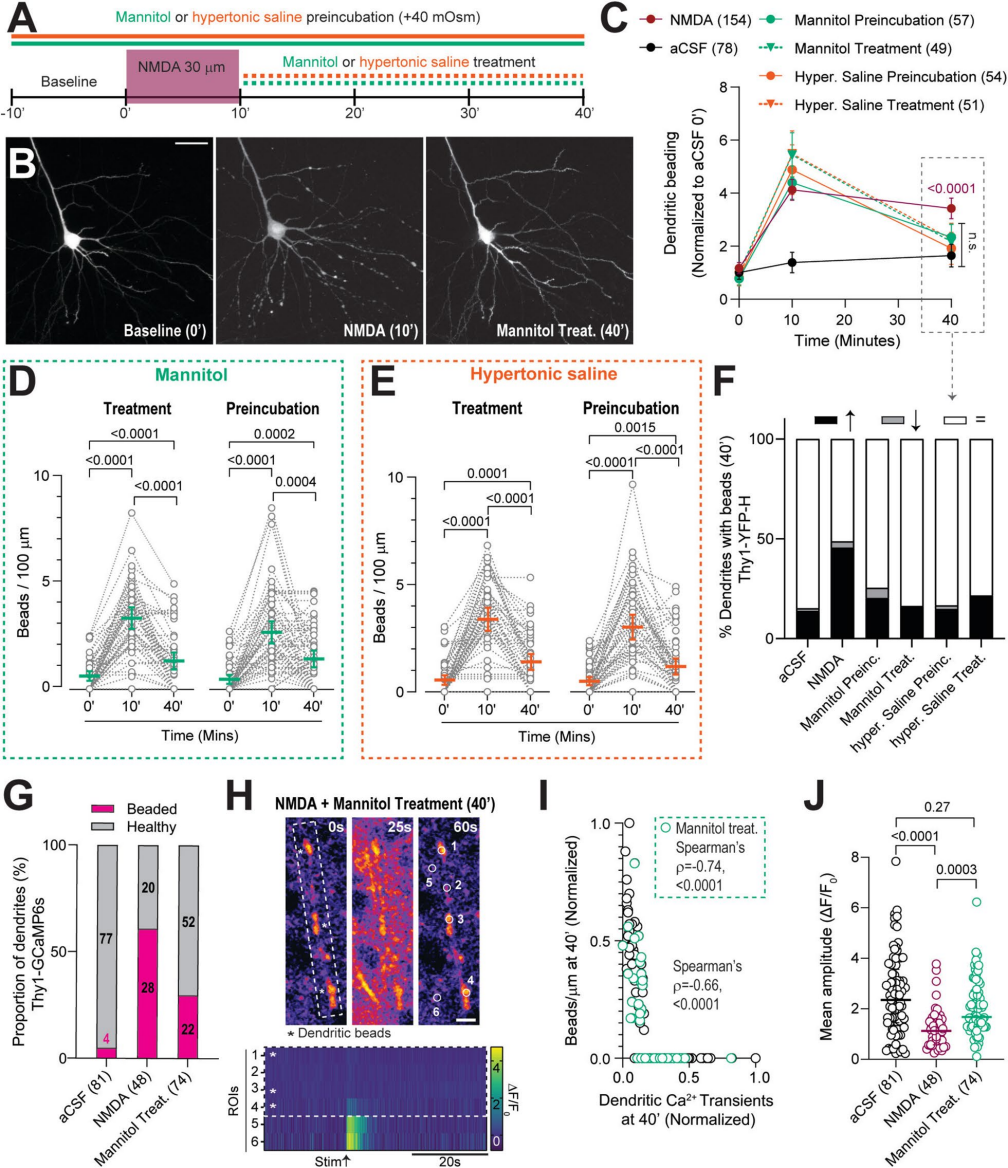
Hyperosmotic interventions resolve dendritic beads and restore dendritic Ca^2+^ activity. **A**, Experimental design describing preincubation and treatment of hyperosmotic interventions (+ 40 mOsm). **B**, Example images showing the resolution of NMDA-induced dendritic beads at 40′ using mannitol treatment. **C**, Comparison of normalized dendritic bead density under different conditions (Two-way ANOVA with Tukey’s test, Interaction: F(10, 1311) = 10.8, p < 0.0001, at 40′: aCSF vs. NMDA p < 0.0001). **D**, Preincubation and treatment with mannitol led to a partially restored NMDA-induced dendritic beading (Friedman test with Dunn’s test, left: p < 0.0001, n = 4:5:57; right: p < 0.0001, n = 3:4:49). **E**, Similarly, preincubation and treatment with hypertonic saline partially restored dendritic beading (Friedman test with Dunn’s test, left: p < 0.0001, n = 3:4:54; right: p < 0.0001, n = 3:3:51). **F**, Proportion of dendrites with an increase, decrease, or no change in beading at 40′ across multiple conditions (threshold: mean of aCSF 0′ ± SD). Both hyperosmotic interventions significantly reduced the proportion of beaded dendrites (Fisher's exact test, p < 0.0001). **G**, Mannitol treatment significantly reduced the proportion of beaded dendrites at 40′ post-NMDA in Thy1-GCaMP6s slices (Chi-square test, df (44.6, 2), p < 0.0001, numbers represent analyzed dendrites). **H**, Representative images of a beaded dendrite after NMDA + mannitol treatment (P10, > 40′) before, during, and after stimulation (top). Heatmap of Ca^2+^ transients derived from ROIs drawn on the beaded dendrite (ROIs 1–4, * represents ROIs on beads) and adjacent healthy dendrites (ROIs 5, 6), showing interrupted propagation of the Ca^2+^ signal along the beaded dendrite. **I**, Mean amplitudes of dendritic Ca^2+^ transients were inversely correlated with bead density (Spearman’s ρ = − 0.66, p < 0.0001). Green circles represent Ca^2+^ signal from the subset of dendrites treated with mannitol (correlation with bead density (box): Spearman’s ρ = − 0.74, p < 0.0001). **J**, Significant reduction in the mean amplitudes of dendritic transients rescued by mannitol treatment (Kruskal–Wallis statistic with Dunn’s test, p < 0.0001). Data: Median ± IQR. Scale bars: 50 µm for **B**, 10 µm for **H**. See also Video S4

Dendritic beading is thought to be mediated by varicose swelling [36, 67]. We investigated the role of common clinical hyperosmotic interventions (+ 40 mOsm of mannitol or hypertonic saline) in mitigating edema and, consequently, dendritic beading, during early brain development. Hyperosmotic conditions were applied during preincubation (10 min before NMDA) or as treatments after NMDA application, which mimics a more clinical scenario, in Thy1-YFP acute brain slices (Fig. 8A). We found that preincubation and treatment with mannitol or hypertonic saline mitigated NMDA-induced dendritic beading, with no significant difference in beading between hyperosmotic conditions and aCSF alone at 40 min (Fig. 8B, C). Matched analysis of each hyperosmotic condition demonstrated that NMDA-induced dendritic beading significantly decreased under both conditions at 40 min but remained slightly higher than baseline (Fig. 8D, E). Importantly, the proportion of beaded dendrites was significantly reduced at 40 min under all hyperosmotic conditions (Fig. 8F). As both mannitol and hypertonic saline decreased the number of dendritic beads, we chose to evaluate mannitol’s effects on dendritic Ca^2+^ dynamics. Mannitol treatment significantly reduced the proportion of beaded dendrites expressing GCamP6s, similar to our findings with YFP-expressing dendrites (Fig. 8G). Nevertheless, even with mannitol treatment, the dendrites that retained beads showed hindered Ca^2+^ propagation along their length (Fig. 8H, Video S4) and the mean amplitude of the dendritic Ca^2+^ transients was still inversely correlated with bead density (Fig. 8I). However, by decreasing the number of beaded dendrites, mannitol indirectly restored the mean amplitude of dendritic Ca^2+^ transients compared to NMDA-treated slices (Fig. 8J). These data indicate that hyperosmotic treatment reduces dendritic beading and restores dendritic Ca^2+^ activity in developing brain circuits, thereby increasing the proportion of healthy dendrites.

## Discussion

Varicose swelling of the dendritic segments, appearing as an alternating pattern of rounded beads (dendritic beading), has been observed after severe seizures and various types of injury [17, 22, 24, 59]. Excitotoxic dendritic beading can be transient or long-lasting and often represents “dendrotoxicity,” a precursor to neuronal injury following various neurological insults [51, 52]. Most clinical and experimental evidence for dendrotoxicity phenotypes comes from studies involving adults, while the morphological and functional nuances of excitotoxic dendritic beading during early development remain relatively unexplored. Our study demonstrates robust dendritic beading in neocortical and hippocampal neurons after brief NMDAR stimulation and recurrent seizures (status epilepticus) in acute brain slices and awake, behaving mice during early brain development (Figs. 1, 2, 3). We found a uniform spatial distribution of excitotoxic beading in the basal dendrites and the oblique branches or tufts of the apical dendrites (Fig. 4). Unlike observations from adult mice [28, 29, 66], we did not see progressive beading of apical dendrites proximal to the neuronal soma. Dendritic beading during early development was accompanied by dendritic spine loss in acute brain slices and in vivo (Fig. 5). We also found persistently high cytosolic Ca^2+^ levels within the dendritic varicosities and a propagation block of stimulus-evoked dendritic Ca^2+^ responses due to the presence of the beads (Fig. 6). In the hippocampal network, we demonstrated that slices exposed to beading-inducing stimuli exhibited rapid suppression of evoked extracellular field potentials, a slow recovery, and impaired short- and long-term synaptic plasticity (Fig. 7). Finally, different clinically relevant hyperosmotic interventions resolved excitotoxicity-induced dendritic beads and, as a result, facilitated the recovery of dendritic Ca^2+^ responses (Fig. 8). Our results elucidate the phenotype of excitotoxic dendritic beading during early brain development and indicate that excitotoxicity-induced dendritic dysfunction may contribute to memory defects associated with prolonged and intense seizures.

Previous studies have reported inconsistencies in dendritic beading, with the extent of beading likely depending on the duration or severity of excitotoxic insults [22, 49, 59]. Our approach, which involved severe and prolonged recurrent seizures modeled by brief NMDAR stimulation (Fig. 1) and prolonged 4-AP treatment (Fig. 3E, I), produced reliable dendritic beading in neocortical and hippocampal slices of the developing brain, enabling their quantification and the evaluation of common clinical interventions. While it took longer for 4AP to induce statistically significant beading compared to NMDA perfusion, this is most likely due to the sustained Ca^2+^ influx with NMDA, compared to the intermittent influx with 4-AP. Additionally, we demonstrated that brief NMDAR stimulation in awake, behaving young mice also induced robust dendritic beading (Fig. 3A–D), consistent with other in vivo chemoconvulsant models where dendritic beading correlated to seizure severity [22]. Although we used a high NMDA concentration (125 µM), this was necessary for the NMDA to penetrate the brain parenchyma through the intact dura mater. Additionally, although we used semi-automated methods for spine detection and our experiments were not blinded due to the evident neuronal Ca^2+^ activity and beading caused by NMDA and 4-AP, incorporating fully automated machine learning approaches in future research could further enhance rigor. Nevertheless, our models, which yielded robust and reproducible dendritic beading, enabled us to characterize this phenomenon in the developing brain.

Adult mouse studies suggest that the influx of extracellular Ca^2+^ through excessive glutamate receptor activation is necessary for the formation of dendritic beads [24, 28, 29]. Although we did not assess the necessity of extracellular Ca^2+^ in early-life dendritic bead formation by altering extracellular Ca^2+^ concentrations, we observed persistently high Ca^2+^ (GCaMP6s) signals within the dendritic beads. The dendritic bead densities also decreased after NMDA receptor antagonism, supporting adult mice results [24]. Additionally, the influx of water and chloride through cation-chloride cotransporters and other transporters is necessary to induce dendritic beading [67, 84], similar to what is observed in neuronal somatic swelling [62, 75]. Compared to hypertonic saline, mannitol can reduce seizures by decreasing neuronal chloride accumulation [18]. Interestingly, one might expect that hypertonic saline would exacerbate dendritic beading due to a greater gradient of sodium and chloride entering the dendrites, particularly since the canonical chloride (and sodium) importer NKCC1 is expressed at this age [38]. However, this was not the case as both mannitol and hypertonic saline decreased dendritic beading. Importantly, we observed similar effects whether the hyperosmotic therapies were administered before or after NMDA perfusion.

We observed differences in how dendrites swell in response to excitotoxic injury between the developing and adult brains. Studies in adult hippocampal slices indicate that brief NMDAR stimulation induces beading in the basal dendrites and distal branches of apical dendrites, followed by delayed and progressive beading in the proximal sections of apical dendrites, along with persistent suppression of evoked hippocampal field potentials [28, 29]. In contrast, although applying the same brief NMDAR stimulation protocol on developing brain slices (P11–13) induced beading of distal and oblique branches and tufts, it did not lead to delayed, progressive beading in proximal apical dendrites up to 70 min after NMDA (Fig. 4). Additionally, in contrast with adult brain slices, the suppressed evoked-field responses after NMDAR stimulation gradually recovered to about 50% of the baseline responses within 70 min in a subset of slices from developing hippocampi (Fig. 7). These results may indicate different susceptibilities to “dendrotoxicity” induced by excessive glutamate receptor stimulation between acute brain slices from developing and adult brains. Age-related changes in energy metabolism may explain differences in dendritic susceptibility [21, 59, 60]. Additionally, differences in cytoskeletal protein dynamics may also contribute. The mixed polarity of microtubules and the expression of MAP2 are critical, particularly for the development and stability of apical dendrites [10, 23]. Loss of mixed microtubule polarity in mature neurons [6, 39], along with the loss of MAP2 function [11, 33], may contribute to increased susceptibility to apical dendrite beading. Also, microglial involvement in seizure-induced dendritic bead resolution [15] suggests a potential difference in microglial responses to dendritic injury between adult and developing brains. However, the precise molecular mechanisms are unknown, and more research is needed to investigate the age-related vulnerability to dendritic beading, particularly in the apical dendrites.

Early life exposure to prolonged seizures is linked to intellectual disabilities and cognitive impairments later in life [3, 13, 40, 77]. Excitotoxicity-induced dendritic beading results in a significant loss of dendritic spines. This loss has been observed in the neocortex and hippocampus of pathological specimens from both human epilepsy patients and animal models [12, 32, 37, 72]. Similar findings have also been reported in other forms of brain excitotoxicity, including spreading depolarization, hypoxic-ischemic insults, and brain injury [53, 67, 88]. Dendritic spines serve as the anatomical loci for glutamatergic synaptic inputs onto neurons and are critical for the expression of synaptic plasticity and learning [14, 69]. The “critical period” for synaptic plasticity during early development is characterized by a prevalence of “silent” synapses with only NMDA receptors, which can encode information (unsilenced) after activity-dependent insertion of AMPA receptors [31, 35]. Early developmental seizures can cause premature unsilencing of these synapses, thereby impairing activity-dependent plasticity [70]. Our results support an alternative mechanism: spine loss triggered by excitotoxicity or seizure-induced dendritic beading [49, 61, 81] would mimic premature spine elimination, impairing synaptic plasticity [64, 68]. This process may serve as an additional pathological substrate, alongside dendritic beading, for memory deficits and other cognitive impairments associated with early-life seizures [16]. Therefore, addressing dendritic beading and related spine loss may serve as therapeutic targets to prevent neurocognitive sequelae linked to early-life excitotoxicity.

We observed persistently elevated cytosolic Ca^2+^ levels within the dendritic beads. Typically, activity-dependent neuronal Ca^2+^influx leads to only a transient rise in its concentration due to rapid uptake by the endoplasmic reticulum and mitochondria, and other extrusion mechanisms [5, 26, 57]. This Ca^2+^ uptake is critical for preventing runaway excitation at dendritic spines [54] and may fail due to altered and prolonged mitochondrial depolarization [24, 63]. A co-occurrence of depolarized mitochondria has been observed in regions containing dendritic beads in cultured neurons, suggesting a potential mechanism for the persistently elevated localized Ca^2+^ levels in these areas [21]. We also found that dendritic beads suppress evoked Ca^2+^ transients, as regions containing these beads hinder the propagation of Ca^2+^ signals along the dendritic segments. We hypothesize that this may result from abnormally high localized Ca^2+^ levels, resulting from persistent membrane depolarization, or altered extrusion or buffering mechanisms at dendritic beads. However, the exact mechanism remains unknown. Nonetheless, the outcome is altered dendritic transmission, which impacts signal processing.

In summary, we demonstrated that long-lasting dendritic beading and spine loss following early-life excitotoxicity, including seizures, harm neural processing and synaptic plasticity in the developing brain. These dendritic changes, if not reversed, can lead to cognitive defects. Hypertonic treatment reverses a significant portion of dendritic beading. Future studies into the molecular mechanisms that mediate dendritic beading and related spine loss during early brain development could identify new therapeutic targets to prevent cognitive defects related to early-life excitotoxicity.

## Supplementary Information

Additional file1

Additional file2

Additional file3

Additional file4

Additional file5

## Data Availability

The datasets and code used for image processing and data analysis in the current study have not been deposited in a public repository but are available on request. Further information and requests for resources and reagents should be directed to and will be fulfilled by the lead contact and corresponding author, Dr. Joseph Glykys (joseph-glykys@uiowa.edu).

## Declarations

### Resource availability

This study did not use or generate any new, unique reagents. The datasets and code used for image processing and data analysis in the current study have not been deposited in a public repository but are available on request. Further information and requests for resources and reagents should be directed to and will be fulfilled by the lead contact and corresponding author, Dr. Joseph Glykys (joseph-glykys@uiowa.edu).
