# Supplementary material for: Dendritic beading during early brain development impairs signal transmission and synaptic plasticity
Source: Acta Neuropathol Commun. 2025 Oct 6;13:212. doi: 10.1186/s40478-025-02123-8 (PMC12502544; doi:10.1186/s40478-025-02123-8)
Supplement: Supplementary file 1 — Additional file1 [file 40478_2025_2123_MOESM1_ESM.docx]

**Supplementary information**

**Table S1: Description of data parameters for each figure.**

|  | **Age** | **Brain regions** | **Experimental technique** | **Mouse strains** |
| --- | --- | --- | --- | --- |
| **Fig. 1** | P11-13 | 1A, C: Neocortex.  1B: Whole slice | 1A, B: Slice 2P imaging, Field electrophysiology.  1C1C: mesoscopic fluorescent imaging. 1D: Field electrophysiology | Thy1-GCaMP6s  C57BL/6JC57BL/6J |
| **Fig. 2** | P11-13 | 2A-C: Neocortex  2D-F: Hippocampus | Slice 2P imaging | Thy1-YFP |
| **Fig. 3** | 3A-D: P14-17  3E-I: P11-13 | Neocortex | 3A-D: *in vivo* 2P imaging  3E-I: Slice 2P imaging | Thy1-GCaMP6s, Thy1-YFP |
| **Fig. 4** | P11-13 | Neocortex | Slice 2P imaging | Thy1-YFP |
| **Fig. 5** | 5A-D: P11-13  5E-G: P14-17 | Neocortex | 5A-D: *in vivo* 2P imaging  5E-G: Slice 2P imaging | Thy1-YFP |
| **Fig. 6** | P11-13 | Neocortex | Slice 2P imaging | Thy1-GCaMP6s, Thy1-YFP |
| **Fig. 7** | P14-19 | Hippocampus | Field electrophysiology | C57BL/6JC57BL/6J |
| **Fig. 8** | P11-13 | Neocortex | Slice 2P imaging | Thy1-GCaMP6s, Thy1-YFP |


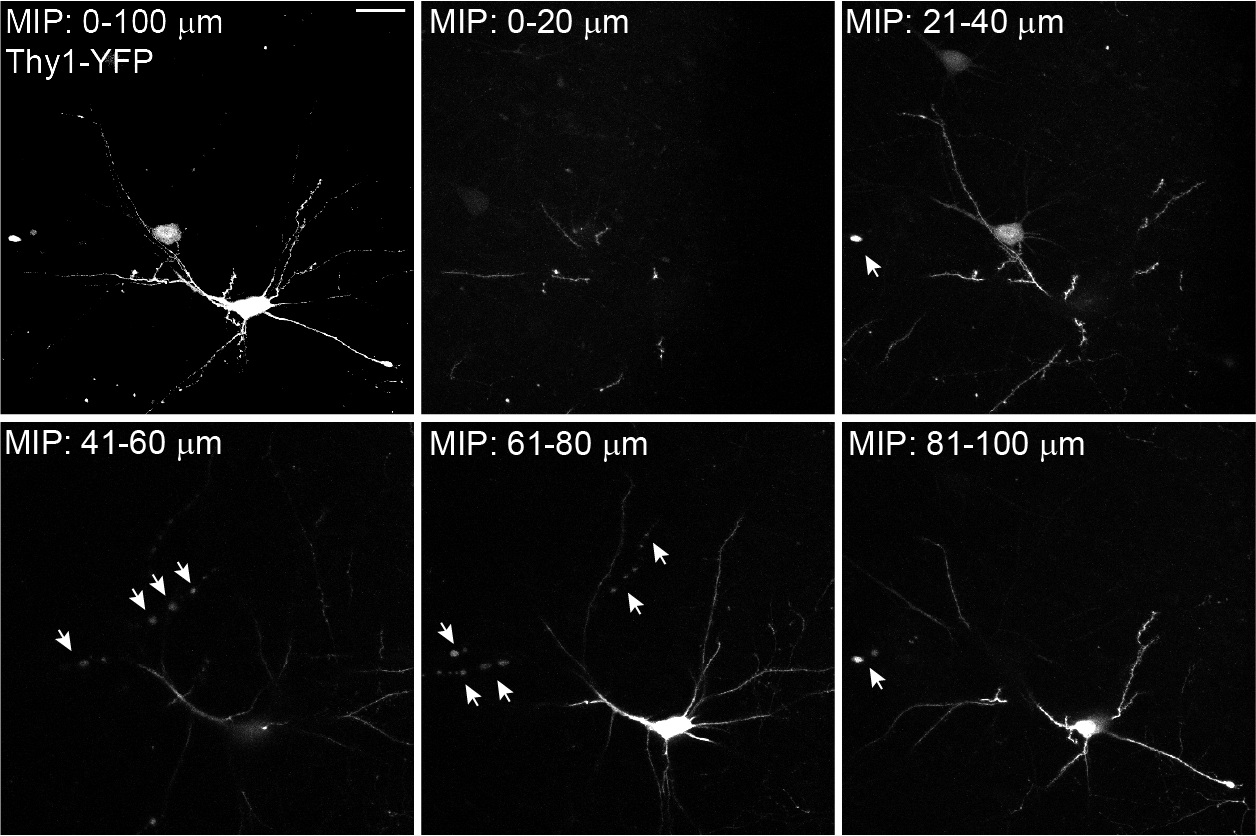


**Fig. S1. Baseline dendritic beads in acute Thy1-YFP slices (P11-13).**

*Top Left*: Representative maximum intensity projection (MIP) of a Z-stack (100 µm depth) showing a neuronal soma and distribution of dendritic branches at baseline (Thy1-YFP). *Right and bottom panels*: MIPs of 10 mm thick sections of the stack showing the presence of dendritic beads at baseline and their distribution across depth. Scale bar: 50 µm.


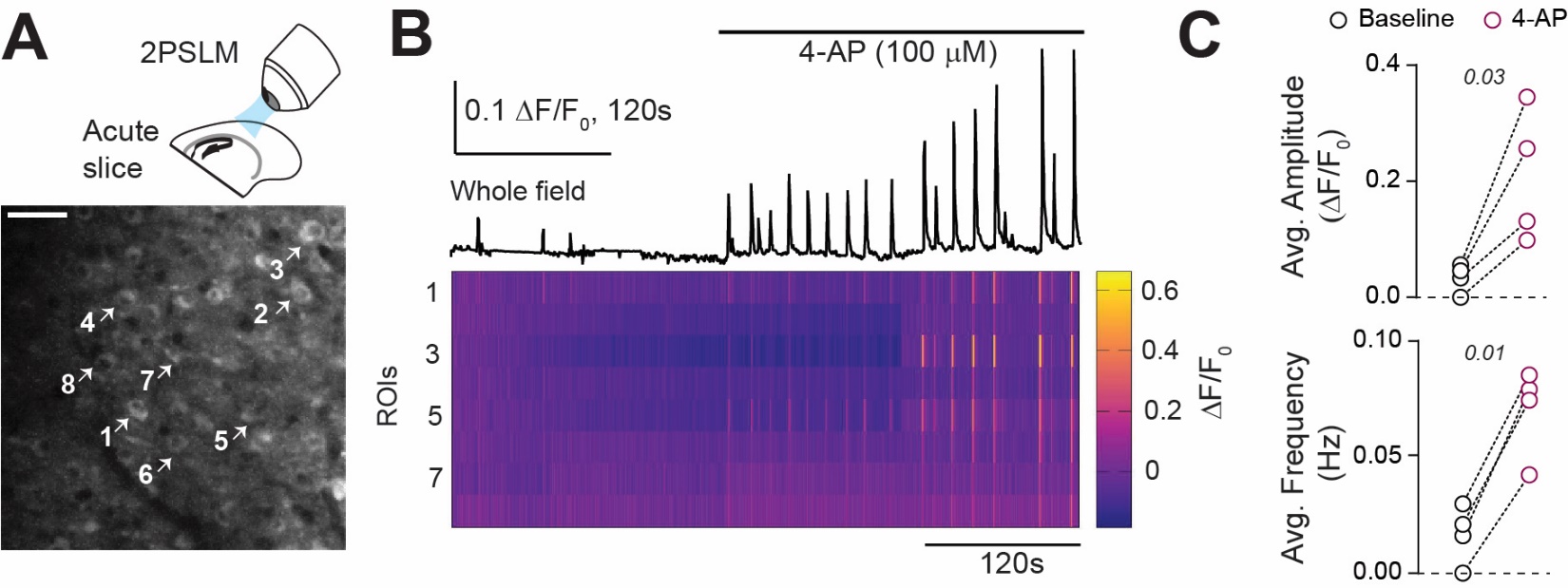


**Fig. S2. Difference between spontaneous and 4-AP-induced synchronous neuronal activity in acute, Thy1-GCaMP6s slices (P11-13).**

**A,** Experimental design and a representative image showing neuronal ROIs (arrows). **B**, Whole field Ca^2+^ transients (*top*) and heatmap showing synchronous neuronal Ca^2+^ transients during baseline and 4-AP treatment. **C**, Significant increase in average amplitudes and frequencies of whole field Ca^2+^ transients of acute slices after the application of 4-AP (amplitude (t=3.7, dt=3): p=0.03, frequency (t=12.5, dt=3): p=0.01; paired t-test). Scale bar: 50 µm.


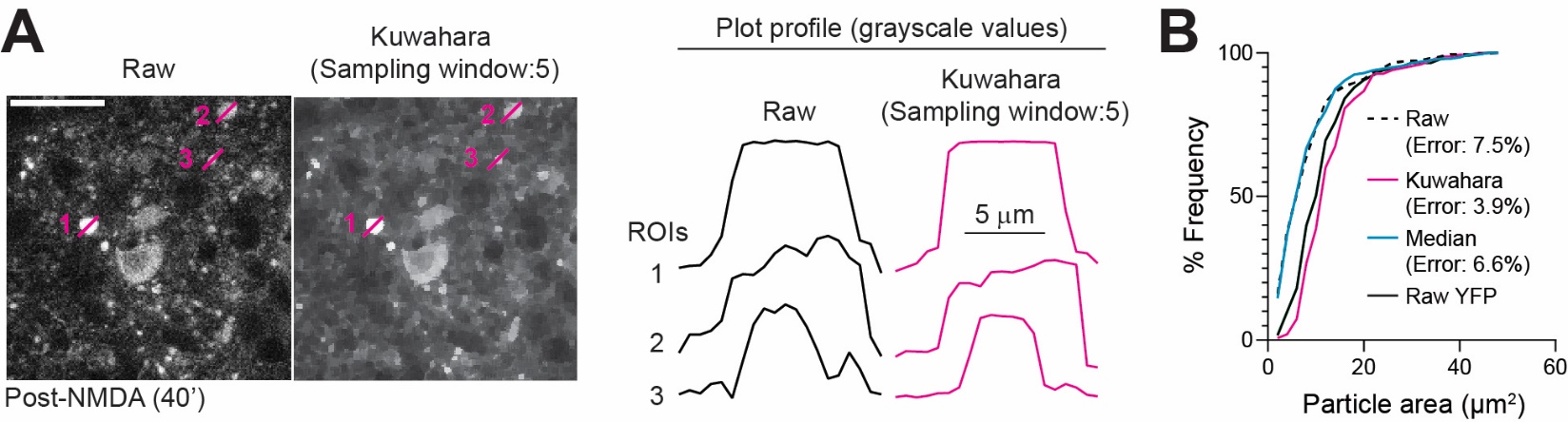


**Fig. S3. Image smoothing with Kuwahara filter for bead detection in Thy1-GCaMP6s slices.**

**A,** Maximum intensity projections (MIPs, Thy1-GCaMP6s) of raw Z-stacks acquired 40’ post-NMDA and the same stack smoothened with Kuwahara filter (Sampling window: 5). Plot profiles of grayscale values derived from 14 µm linear ROI’s show differences in signal distribution across dendritic beads using (*right*). **B**, Frequency distribution plots showing different distribution areas (µm^2^) of beads detected using analyze particle function (ImageJ, size: 2-50, circularity: 0.75 to 1) between raw Thy1-GCaMP6s and Thy1-YFP images, and Thy1-GCaMP6s images filtered with median (Kernel: 2) and Kuwahara filters (Sampling window: 5). Smoothing with Kuwahara filter resulted in a reduced number of particle detections with smaller areas (suggesting reduced noise) with lower false detection rate or % Error. The distribution of particles detected with the Kuwahara filter resembles the distribution of detections acquired using Thy1-YFP slices. Scale bar: 50 µm.

**Movie S1. Mesoscopic Ca^2+^ imaging during brief NMDAR perfusion. (Related to Fig. 1).**

Synchronous neuronal Ca^2+^ activity following brief NMDAR stimulation was acquired using Thy1-GCaMP6s slices. The video shows neuronal Ca^2+^ activity for the entire slice at baseline, followed by synchronous Ca^2+^ spikes (related to increased FFT power) and a sustained high Ca^2+^ signal (related to the DC shift) evoked by NMDAR stimulation. Data were acquired at ~20 Hz. A median filter was applied (radius:2) for smoothing. Playback speed: 120×. Scale bar (white): 1 mm.

**Movie S2. *In vivo* multiphoton imaging of an awake, behaving mouse during early development at baseline and during NMDA application. (Related to Fig. 3).**

Multiphoton imaging of neuronal Ca^2+^ activity (*left*) and corresponding behavior/locomotion (right) was acquired from an awake, head-fixed developing mouse pup (P15), during baseline and NMDAR stimulation. The video shows neuronal Ca^2+^ transients synchronized with locomotion at baseline. NMDAR stimulation induced a robust increase in neuronal Ca^2+^ activity with a complete absence of locomotion. The 2PSLM data were acquired at ~29 Hz (512×512 resolution, 32× magnification), and the behavioral data at ~20 Hz. 2PSLM images were smoothed by a median filter (radius:2). Playback speed: 48×. Scale bar (white): 50 μm.

**Movie S3. Dendritic beads impede the propagation of Ca^2+^ transients. (Related to Fig. 6).**

Stimulus-evoked Ca^2+^ transients in dendrites with or without beads. The video shows raw data (*left*) and ΔF/F_0_ projection (*right*), demonstrating the absence of stimulus-evoked Ca^2+^ responses in dendritic beads (*), which halts propagation. Notice the lack of ΔF/F_0_ increase (stay black) in the beads during stimulation. Images were smoothed by a median filter (radius:2). Acquired at ~29 Hz (512×512 resolution, 64× magnification). Playback speed: 12×. Scale bar (white): 10 μm.

**Movie S4. Mannitol does not reverse evoked Ca^2+^ activity in dendrites containing beads (Related to Fig. 8).**

Stimulus-evoked Ca^2+^ transients in healthy and beaded dendrites, during hyperosmotic conditions (mannitol). The video shows raw data (*left*) and ΔF/F_0_ projection (*right*), indicating that beaded dendrites lack stimulus-evoked Ca^2+^ responses. The ROI indicates the example dendrite displayed in Fig. 8H. Notice the lack of ΔF/F_0_ increase (stay black) in the beads during stimulation. Images were smoothed by a median filter (radius:2). The data were acquired at ~29 Hz (512×512 resolution, 32× magnification). Playback speed: 12×. Scale bar (white): 50 μm.
